# X-ray Crystallographic Structure and Oligomerization of *Gloeobacter* Rhodopsin

**DOI:** 10.1038/s41598-019-47445-5

**Published:** 2019-08-02

**Authors:** Takefumi Morizumi, Wei-Lin Ou, Ned Van Eps, Keiichi Inoue, Hideki Kandori, Leonid S. Brown, Oliver P. Ernst

**Affiliations:** 1 Department of Biochemistry, University of Toronto, Toronto, Ontario M5S 1A8 Canada; 2 Department of Molecular Genetics, University of Toronto, Ontario, M5S 1A8 Canada; 3 The Institute for Solid State Physics, University of Tokyo, Kashiwa, Chiba 277-8581 Japan; 4 Department of Life Science and Applied Chemistry, Nagoya Institute of Technology, Showa-ku, Nagoya 464-8555 Japan; 5 OptoBioTechnology Research Center, Nagoya Institute of Technology, Showa-ku, Nagoya 464-8555 Japan; 6 Department of Physics, University of Guelph, Guelph, Ontario N1G 2W1 Canada

**Keywords:** X-ray crystallography, Molecular conformation

## Abstract

*Gloeobacter* rhodopsin (GR) is a cyanobacterial proton pump which can be potentially applied to optogenetics. We solved the crystal structure of GR and found that it has overall similarity to the homologous proton pump from *Salinibacter ruber*, xanthorhodopsin (XR). We identified distinct structural characteristics of GR’s hydrogen bonding network in the transmembrane domain as well as the displacement of extracellular sides of the transmembrane helices relative to those of XR. Employing Raman spectroscopy and flash-photolysis, we found that GR in the crystals exists in a state which displays retinal conformation and photochemical cycle similar to the functional form observed in lipids. Based on the crystal structure of GR, we selected a site for spin labeling to determine GR’s oligomerization state using double electron–electron resonance (DEER) spectroscopy and demonstrated the pH-dependent pentamer formation of GR. Determination of the structure of GR as well as its pentamerizing propensity enabled us to reveal the role of structural motifs (extended helices, 3-omega motif and flipped B-C loop) commonly found among light-driven bacterial pumps in oligomer formation. Here we propose a new concept to classify these pumps based on the relationship between their oligomerization propensities and these structural determinants.

## Introduction

Many organisms utilize light-driven transporter proteins to capture light for energy conversion or cell signaling. Rhodopsins are photo-receptive proteins which share a similar overall structure, composed of seven transmembrane α-helices (7-TM), and have the chromophore retinal covalently bound to a lysine residue on TM7 *via* a protonated Schiff base. Light absorption by rhodopsin induces isomerization of a single double bond of the retinal’s polyene chain, which triggers structural changes in the protein moiety to perform vital biological functions^1^. Based on primary amino acid sequences, rhodopsins are classified into two groups, type-1 for microorganisms and type-2 for animals^2^, with predominantly all*-trans* → 13-*cis* and 11-*cis* → all-*trans* retinal isomerization, respectively.

**Figure 1:**
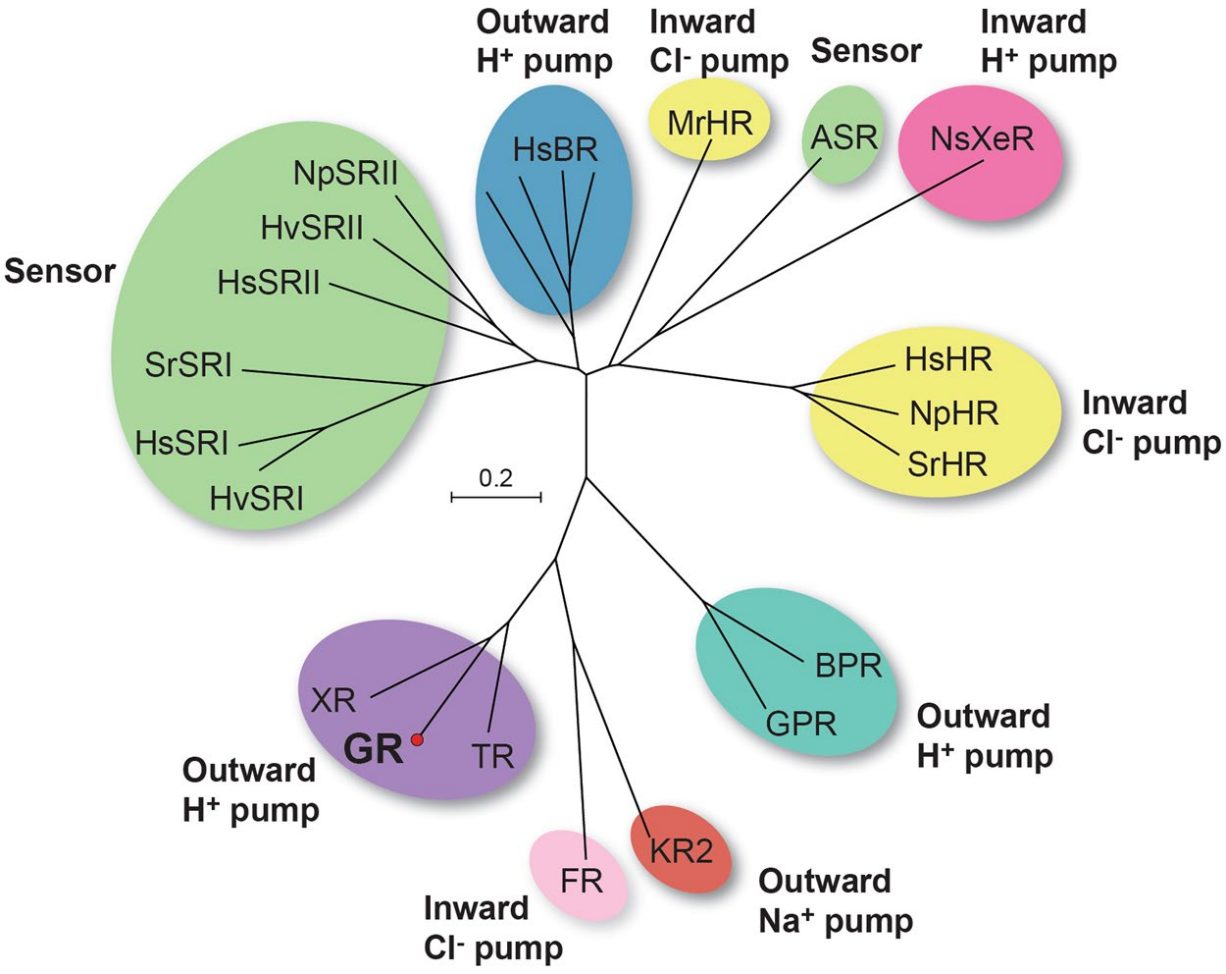
Phylogenetic tree of microbial rhodopsins from *Archaea* and *Eubacteria*, representing the phylogenetic relationship between *Gloeobacter* rhodopsin and related proteins. HsBR, bacteriorhodopsin from *H*. *salinarum*; HvSRI, HsSRI, SrSRI, sensory rhodopsin I from *Haloarcula vallismortis*, *H*. *salinarum*, and *Salinibacter ruber*; HsSRII, HvSRII, NpSRII, sensory rhodopsin II from *H*. *salinarum*, *Haloarcula vallismortis*, *Natronomonas pharaonis*; MrHR, *Mastigocladopsis repens* halorhodopsin; ASR, *Anabaena* sensory rhodopsin; NsXeR, *Nanosalinarum sp* xenorhodopsin; HsHR, NpHR, SrHR, halorhodopsin from *H*. *salinarum*, *N*. *pharaonis*, and *S*. *ruber*; BPR, blue-absorbing proteorhodopsin; GPR, green-absorbing proteorhodopsin; KR2, sodium-pumping rhodopsin from *Krokinobacter eikastus*; FR, chloride-pumping rhodopsin from *Fulvimarina pelagi*; TR, thermophilic rhodopsin from *Thermus thermophilus*; GR, rhodopsin from *Gloeobacter violaceus* PCC 7421; XR, xanthorhodopsin from *S*. *ruber*. The evolutionary history was inferred using the Neighbor-Joining method^64^. The optimal tree with the sum of branch length = 11.29080174 is shown. The tree is drawn to scale, with branch lengths in the same units as those of the evolutionary distances used to infer the phylogenetic tree. The evolutionary distances were computed using the Poisson correction method^65^ and are in the units of the number of amino acid substitutions per site. The analysis involved 23 amino acid sequences. All positions containing gaps and missing data were eliminated. There was a total of 189 positions in the final dataset. Evolutionary analyses were conducted with MEGA7^66^.

Microbial rhodopsins are found in *Archaea*, *Bacteria*, lower eukaryotes and viruses^3^. They have a variety of functions, such as light-driven ion pumps (bacteriorhodopsin in *Archaea*; BR), light-gated ion channels (channelrhodopsin in green algae, ChR), and photosensors (sensory rhodopsins I and II in *Archaea* and *Bacteria*; SRI and SRII) (Fig. 1)^4^. Although monomeric forms of microbial rhodopsins seem to be the functional unit^5^, several studies have revealed that microbial rhodopsins mostly exist as oligomers in the native membrane environment^6,7^. Previous studies have suggested functional significance of microbial rhodopsin oligomerization^8,9^, and even functional switch from proton to sodium pumping for KR2^10^; however, the molecular basis that defines the specific oligomeric assemblies for different rhodopsins are unclear and remain to be elucidated^11^. There are a number of biophysical methods to characterize oligomerization^12^. For example, specific oligomeric assemblies of rhodopsins can be crystallized depending on the crystallization conditions, such as dimeric or trimeric structures of BR^13^, pentameric or hexameric structures of blue-absorbing proteorhodopsin (BPR)^9^, and monomeric or pentameric structures of KR2^10,14^. In addition to X-ray crystallography, circular dichroism (CD) spectroscopy^15,16^ and high-speed atomic force microscopy (AFM)^17,18^ have been successful in determining the oligomeric states of microbial rhodopsins. Moreover, electron paramagnetic resonance (EPR) spectroscopy combined with site-directed spin-labeling is a powerful tool not only for characterizing conformational changes in microbial rhodopsins, but also for intermolecular distance measurements between protomers providing structural insight into oligomer formation of these 7-TM proteins^19,20^.

*Gloeobacter* rhodopsin (GR) is a BR-like light-driven proton pump found in unicellular cyanobacterium *Gloeobacter violaceus* PCC 7421, which lacks thylakoid membranes, and its photosynthetic system co-exists in the cytoplasmic membrane with GR^21^. Previous studies have suggested it is likely that GR could support ATP synthesis and compensate the poor energy production when the chlorophyll-dependent photosynthesis is low^22^. Interestingly, GR has carotenoid binding capability similar to that found for xanthorhodopsin (XR)^23^. With the ability of binding light-harvesting carotenoid antenna, *Gloeobacter* cells can utilize various wavelengths of light for energy conversion. In addition, GR shows under specific conditions an inverted proton flux, an interesting feature that other proton pumps commonly applied for optogenetic studies do not have^24^. From recent comprehensive AFM imaging of microbial rhodopsins, the oligomeric state of GR in the reconstituted lipid membrane was proposed to be pentameric^17^, although a previous report concluded that GR has a pH-dependent monomer-trimer transition in detergent micelles^25,26^.

In this study, we present the X-ray crystal structure of GR and discuss the structural features that characterize the XR-like protein subgroup, to which it belongs (Fig. 1). In addition, we identify pentameric oligomerization of GR in detergent micelles using double electron–electron resonance (DEER) spectroscopy^27^, which we demonstrate to be a powerful tool for determining oligomeric assemblies of microbial rhodopsins. From the combination of present experimental results and previous studies, we propose here a new concept to classify microbial rhodopsins by their oligomeric assemblies based on conserved structural motifs.

## Results and Discussion

### Overall structure of GR

**Figure 2:**
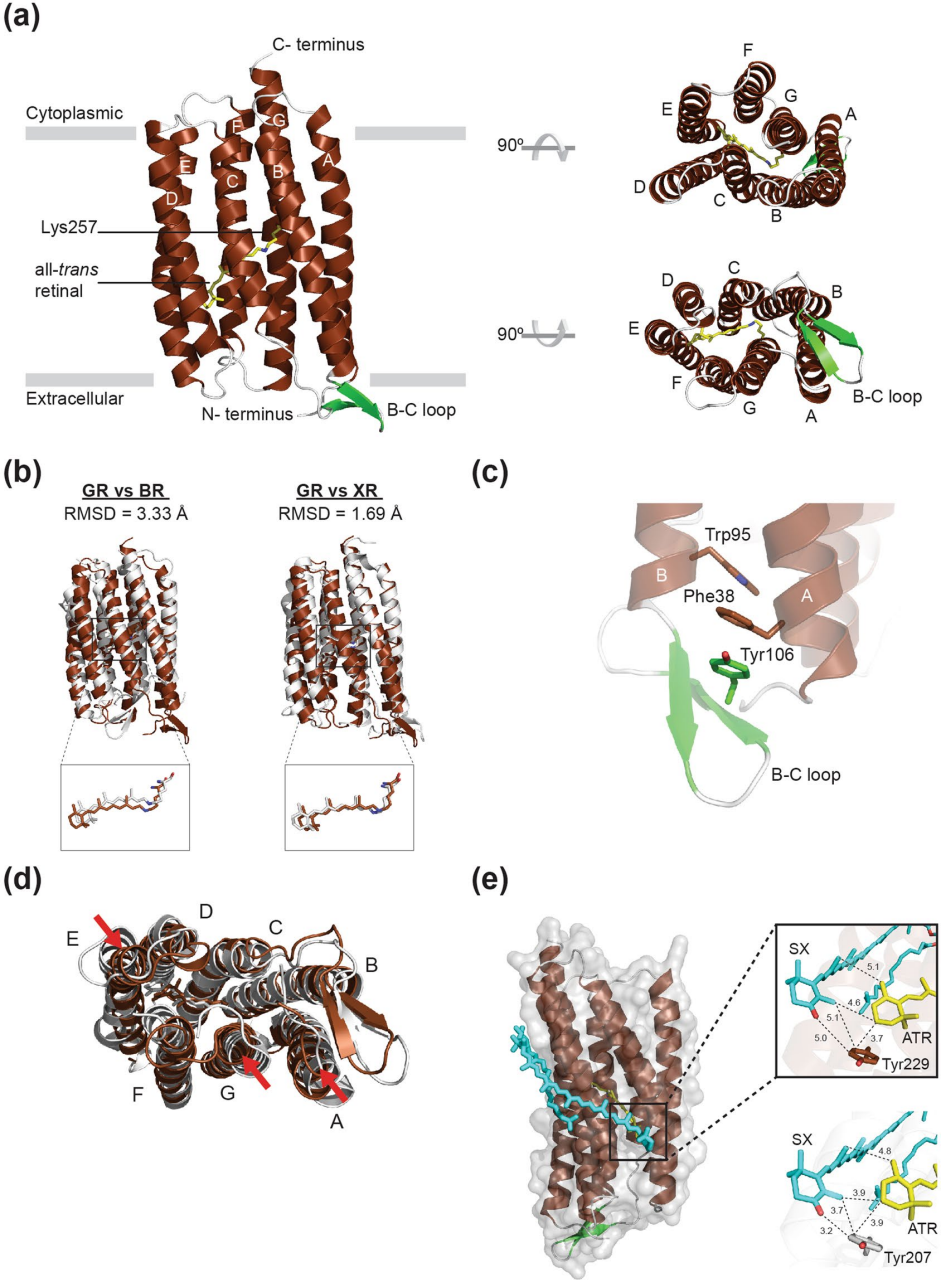
Structural details of GR. (**a**) Overall structure of GR shown in cartoon representation viewed parallel to the membrane (*left*), from intracellular side (*right*, *top*) and from extracellular side (*right*, *bottom*). GR consists of seven transmembrane helices (TM A to TM G, shown in brown) connected by interhelical loops (shown in white) on both sides of the membrane. The β-strands in the B-C loop are shown in green. All-*trans*-retinal, depicted by stick models, is covalently linked to Lys257 via a protonated Schiff base (shown in yellow). (**b**) Superimposed structures of GR (brown) and *H*. *salinarum* BR (white, PDB entry 1C3W), and GR and XR (white, PDB entry 3DDL), respectively. (**c**) 3-omega motif of GR formed by π-stacking interactions of the side chains of aromatic residues in TM A (F38), TM B (W95) and B–C loop (Y106). (**d**) Extracellular side of superimposed GR (brown) and XR (white) structures with the XR-bound SX molecule (cyan). The helix displacements are indicated by red arrows. (**e**) Potential carotenoid binding site in GR. Salinixanthin (SX) from the crystal structure of XR is superimposed onto the structure of GR. The magnified views compare the location of SX and the retinal in GR (*top*) and XR (*bottom*).

Figure 2a shows the crystal structure of the GR dark state. The structure was determined to 2.0 Å resolution (Table 1). Similar to other microbial rhodopsins, the seven transmembrane helices are bundled in a clockwise fashion when observed from the cytoplasmic side, and the all-*trans*-retinal chromophore is covalently bound to the opsin protein moiety via a Schiff base linkage with the Lys257 side chain. When crystal structures of *Halobacterium salinarum* BR (PDB entry 1C3W) and XR (PDB entry 3DDL) were superimposed on GR (Fig. 2b), both root-mean-square deviation (RMSD) values (3.33 with BR and 1.69 with XR) and retinal position indicated that the overall structure of GR is more similar to XR than BR as expected from sequence similarity (Figs 1 and S1).

**Table 1:** Crystal data, data collection, and refinement statistics.

Resolution (Å)	42.79–2.0
Space Group	C 2 2 21
Unit Cell Dimensions	
a (Å)	54.25
b (Å)	129.25
c (Å)	82.65
Alpha (degrees)	90
Beta (degrees)	90
Gamma (degrees)	90
Molecules in the asymmetric unit	1
Solvent content (%)	50
Unique reflections	19313
Completeness for range (%)	96.37
Rcryst (%)	23.03
Rfree (%)	24.44
Rmerge (%)	4.4 (21.6)
R.m.s deviations (bond lengths)	0.009
R.m.s deviations (bond angles)	0.85
Ramachandran outliers (%)	0.82
Clashscore	3.29
Average B-factor	38.72
**Data collected at the Advanced Photon Source (APS)**, **Argonne National Laboratory**	
X-ray source	23ID-D
Detector type	Pilatus 6 M
Collection software	In house software package (JBluIce)
Data indexed, collected, scaled	In house software package, XDS, *Aimless*
Search model PDB ID	3DDL
Model alignment used	Blast web server
Molecular replacement program	*Balbes*
Refinement software	*Phenix*.*Refine* (in the Phenix suite)
Model building software	COOT

On the extracellular side, the B-C loop connecting helices B and C has a distinct antiparallel β-sheet structure similar to most microbial rhodopsins. However, unlike BR which has the β-sheet oriented towards the middle of its 7-TM helical bundle, the β-sheet of GR orients oppositely towards helices A and B. As a result, the tip of the B-C loop is facing towards the periphery of the GR molecule and the central ion-releasing cavity at the extracellular surface is exposed. This change in orientation of the β-sheet was first found in the crystal structure of XR and then successively identified in sodium-pumping rhodopsin (KR2)^14,28^, thermophilic rhodopsin (TR)^29^ and eubacterial light-driven chloride-pumping rhodopsin (ClR)^29,30^. In case of KR2 and ClR, an additional N-terminal helix is accommodated close to the extracellular cavity to “cap” the ion-releasing/uptaking region. Another common structural feature of GR, KR2, TR, and ClR are elongated helices A, B and G. As reported for the ClR structure, GR also shares the “3-omega motif” formed by three nonconsecutive aromatic amino acid side chains of Phe38 (helix A), Trp95 (helix B) and Tyr106 (in B-C loop), tethering the B–C loop in the direction of helices A and B (Fig. 2c). Interestingly, these residues are located exactly on the elongated part of helices A and B of these rhodopsins explaining why other BR-type rhodopsins do not share this motif. In contrast to their functional divergence, this structural motif is strictly conserved among different eubacterial pumps, suggesting the motif is not correlated to the function, but to the specific structural arrangement, such as interprotomer interactions. It should be noted that proteorhodopsins only share the elongated helices but lack the 3-omega motif on it as well as β-sheet structure in B-C loop. Proteorhodopsins possess a significantly shortened B-C loop which is suggested to have a propensity to form a beta turn in NMR studies^31,32^. Therefore, we hereafter refer to these rhodopsins sharing the features of flipped B-C loop, elongated helices, and 3-omega motif as “XR-type” to be discriminated from both BR-type and proteorhodopsins.

Another structural characteristic distinguishing GR from XR and TR is a relatively tight packing of TM helices at the extracellular side (Fig. 2d). When compared with the XR structure (shown in white), helices A, E, and G are slightly tilted inward towards the center of the helical bundle, resulting in a ~6.6 Å closer positioning of extracellular ends of helices E and G, whereas no obvious displacements are observed on the cytoplasmic side. At the extracellular side the displacement of helix A also causes a slight displacement of the interacting B-C loop. The repositioning of helix E might be associated with the binding of an additional photoreceptive carotenoid. XR was originally co-crystalized with salinixanthin (SX), the endogenous carotenoid which is considered to be a potential light-harvesting antenna for XR in *Salinibacter ruber*. GR is also known to bind SX^33^ as well as the endogenous carotenoid echinenone from *G*. *violaceus*, both having a 4-keto headgroup^34^, which should locate close to the headgroup of internal all-*trans*-retinal chromophore for energy transfer. When SX from the XR–SX complex was placed in the analogous position within the GR crystal structure, it fits to the same groove as in XR with a slightly more distant SX headgroup position from retinal chromophore (Fig. 2e). Despite the distant position, however, the SX headgroup in this model showed a subtle steric clash with the inwardly displaced helix E. Given the reported functional binding of SX to GR, helix E might be in the “outward” position as in the XR–SX complex when GR accommodates SX or echinenone. Since GR in our crystals does not have any additional carotenoids, the movement of the extracellular end of helix E is likely to reflect the carotenoid-free structure of GR. It should be noted that other XR-type sodium/chloride pumps, KR2^14^ and ClR^30^ have the same helix E position as GR in the absence of carotenoid, and molecular dynamics simulations on TR showed the inward movement of helix E at higher temperature^29^, suggesting the flexibility of this region.

**Figure 3:**
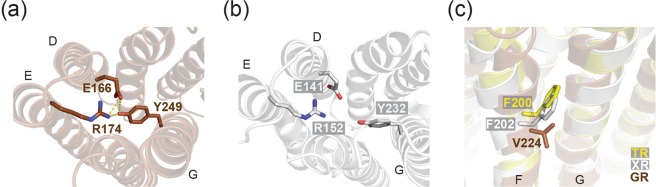
Conserved Glu-Arg salt bridge on the extracellular side of helices D and E; E166-R174 in GR (**a**) and E141-R152 in XR (**b**). (**c**) Steric interference of movement of helices F and G by Phe residues on helix F in XR and TR.

From the crystal structure of GR, the specific residues responsible for controlling the distance between helices E and G could be identified. First, there is a conserved Glu-Arg pair (Glu166-Arg174 in GR, Glu141-Arg152 in XR) on the extracellular side of helices D and E that can electrostatically interact to modulate the relative position of these helices (Fig. 3a). In addition, another conserved tyrosine residue on helix G of GR (Tyr249) participates in this interaction by forming a hydrogen bonded network with Glu166, whereas the corresponding tyrosine residues of XR and TR do not (Fig. 3b). Furthermore, we found a steric constraint presented by a Phe residue in XR and TR, at the position homologous to Val224 on helix F of GR, that could interfere with the movement of helices F and G (Fig. 3c). Located between helices F and G, the bulky side chain of phenylalanine provides (in case of XR and TR) a steric constraint on helix G so that it cannot tilt inwardly as it does in GR. As a result of these interactions, helices E and G come closer in GR but not in XR and TR.

### Structure of retinal binding pocket and the proton transport pathway of GR

**Figure 4:**
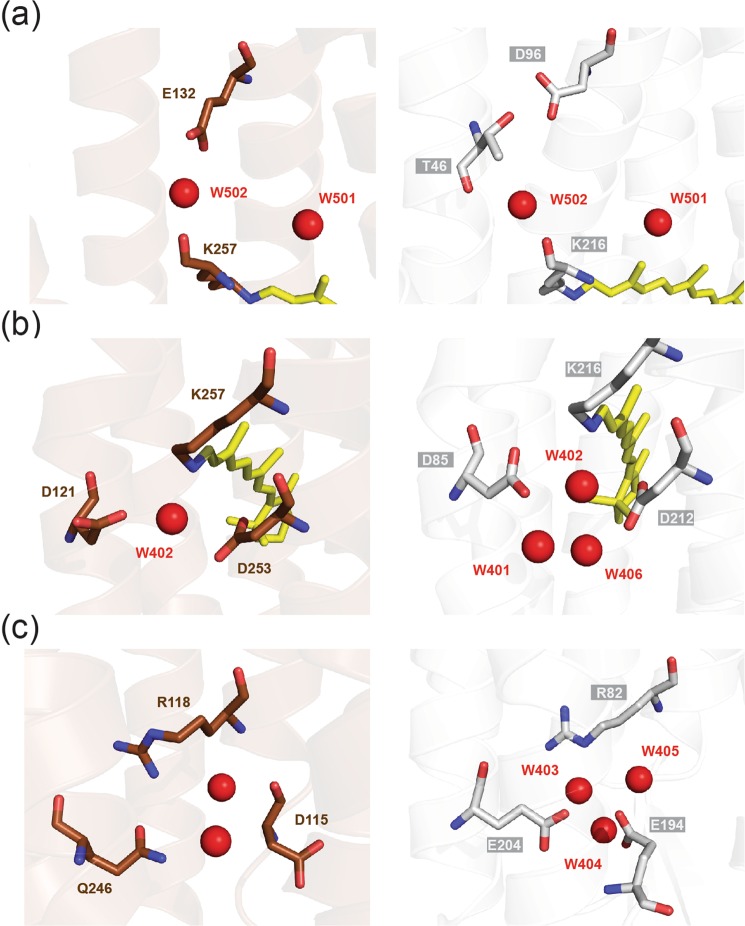
Comparison of three internal cavities in proton transporting pathway of GR (brown) and BR (white). (**a**) Cytoplasmic side of the Schiff base region. (**b**) Extracellular side of the Schiff base region. (**c**) Cavities near the extracellular surface.

When it comes to the proton transport pathway, including the retinal binding pocket, GR has an organization more similar to XR than BR. The three possible regions of hydrogen-bonded network involving the side chains of polar residues and the observed water molecules in the pathway are shown in Fig. 4. It should be noted that the number of water molecules observed to participate in the network depends on the resolution of crystal structures as well as the hydration itself. Therefore, we used water molecules of BR structures that had been confirmed to exist in the internal pathway (wat-401 to 406, 501 and 502) to compare with waters clearly observed in the GR structure (Fig. S2).

First, in the cytoplasmic region of GR, the functionally important water molecules (wat-501, 502) are conserved in both XR and BR. In BR, a proton donor Asp96 is connected to wat-502 through a hydrogen-bonded network via Thr46, whereas the corresponding putative proton donor in GR and XR is replaced by Glu (Glu132 and Glu107, respectively) and interacts directly with wat-502 (Fig. 4a). This structure explains the previous observation of the long-range structural perturbation of Glu132 upon retinal photoisomerization at 77 K^35^. In contrast, the location and hydrogen-bonded network involving wat-501 in GR and XR are almost the same as in BR.

Next, on the extracellular side of the Schiff base of GR, only one water molecule (wat-402 in XR) is mediating the hydrogen-bonded network among Asp121, Asp253 and retinal Schiff base, whereas BR has a water triad (wat-401, 402 and 406) among the corresponding Asp85, Asp212 and the Schiff base (Fig. 4b). Since the water triad in BR has been repeatedly found in structures with comparable resolution (2.0 Å to 2.3 Å, PDB entries 5BR5 and 1IW6), it is likely that GR and XR accommodate only one water molecule instead of the triad. In fact, the side chain of Asp253 in GR comes closer to other residues narrowing the cavity to establish this simplified network with a single water molecule. This water molecule probably forms a strong hydrogen bond with the counterion of the Schiff base as reported by low-temperature FTIR spectroscopy^35^. In addition, similar to XR, GR also has the Asp121-His87 pair as the counterion (Asp85 in BR) of the Schiff base, confirming it is a conserved feature in XR-type proton pumps (XR/GR/TR) as well as proteorhodopsins (BPR/green-absorbing proteorhodopsin, GPR/*Exiguobacterium sibiricum* rhodopsin, ESR). The linkage between the titratable Asp-His pair and the Schiff base would explain the pH dependent shift of the absorption maximum in GR and the GPR dark state spectrum^25,36^.

Lastly, in the extracellular region where the proton release during the photocycle occurs, only two water molecules are found in GR compared with three water molecules in BR (called wat-403, 404 and 405). Also, the surrounding polar residues for this cavity (Asp115, Arg118 and Gln246 in GR) are connected more directly than in BR, accommodating fewer water molecules (Fig. 4c). The most prominent difference found in GR (also in XR/TR) is the lack of the Glu194/Glu204 pair of BR. The hydrogen-bonded network including the Glu194/Glu204 pair and water molecules wat-403 to 405 has been considered to be responsible for the proton release from the extracellular side in the BR photocycle in combination with the rearrangement of the Arg82 side chain^37,38^. XR-type proton pumps lack this Glu/Glu pair, and the glutamate at position 204 is replaced by glutamine (Gln246 in GR). By all these changes, GR establishes a new hydrogen-bonded network connecting Arg118, Gln246, and Asp115 (Fig. 4c). Since Gln246 cannot serve as the proton donor, it is reasonable to assume that Asp115 could take over the proton release function at the extracellular surface instead of the Glu194/Glu204 pair employed in BR. However, confirmation with mutagenesis would be required. The homologous aspartic acid residue is also conserved in XR and TR but not in proteorhodopsins, although they lack the Glu/Glu pair. Instead, another glutamate on helix D, Glu124 in BPR participates in the hydrogen-bonded network, locating the position near to the Glu194 of BR. As mentioned in the previous section, this glutamate in XR-type proton pumps is tightly associated with arginine on helix E (Glu166-Arg174 salt bridge in GR, Fig. 3a) and therefore would not participate in the network. Interestingly, ESR lacks the homolog of Glu194, but retains Glu204 of BR as Glu214.

### Raman spectroscopy and time-resolved laser spectroscopy

Raman spectroscopy and time-resolved laser spectroscopy in the visible range were performed to probe the GR dark state and the photocycle in the crystals (Fig. S3). The plate-like crystals were grown in bicelles at pH 3.4 in the dark (see below) and then harvested for spectroscopic studies. The Raman bands of C=C and C–C stretching vibrations (compare the position of the peak at 1529 cm^−1^ for the former and the ratio of the peaks at 1200 and 1170 cm^−1^ for the latter) in crystals look very similar to those in proteoliposomes at pH 9 but not at pH 3. This suggests that a form of GR with retinal configuration and environment similar to those of the alkaline proton-pumping state in lipids is expected to be found in crystals (Fig. S3a). This assumption is supported by the photocycle kinetics of crystalline GR probed by time-resolved laser spectroscopy in the visible range (flash-photolysis). The photocycle of the alkaline proton-pumping form of GR in lipids is dominated by the late red-shifted O intermediate detectable at 620 nm (Fig. S3b), with strongly pH-dependent kinetics^39^. As reported previously, such accumulation of the late red-shifted O intermediate is diminished when the Schiff base carboxylic counterion is neutralized either by mutation or by protonation at low pH not only in GR, but also in homologous proton pumps XR and PR^40,41^, which correlates with the disappearance of proton transport and the respective M intermediates. Figure S3b shows laser light-induced difference light absorption kinetics of crystalline GR measured at 620 nm compared with those of liposome-reconstituted GR measured earlier^39,42^. The strong accumulation of the red-shifted intermediate and the kinetics of its rise and decay suggests that the photocycle of crystalline GR is similar to that of the alkaline form of GR in lipids at near neutral pH, further confirming similarity of crystalline GR to the functional state of GR in membranes.

### pH-dependent color transition and oligomer formation

**Figure 5:**
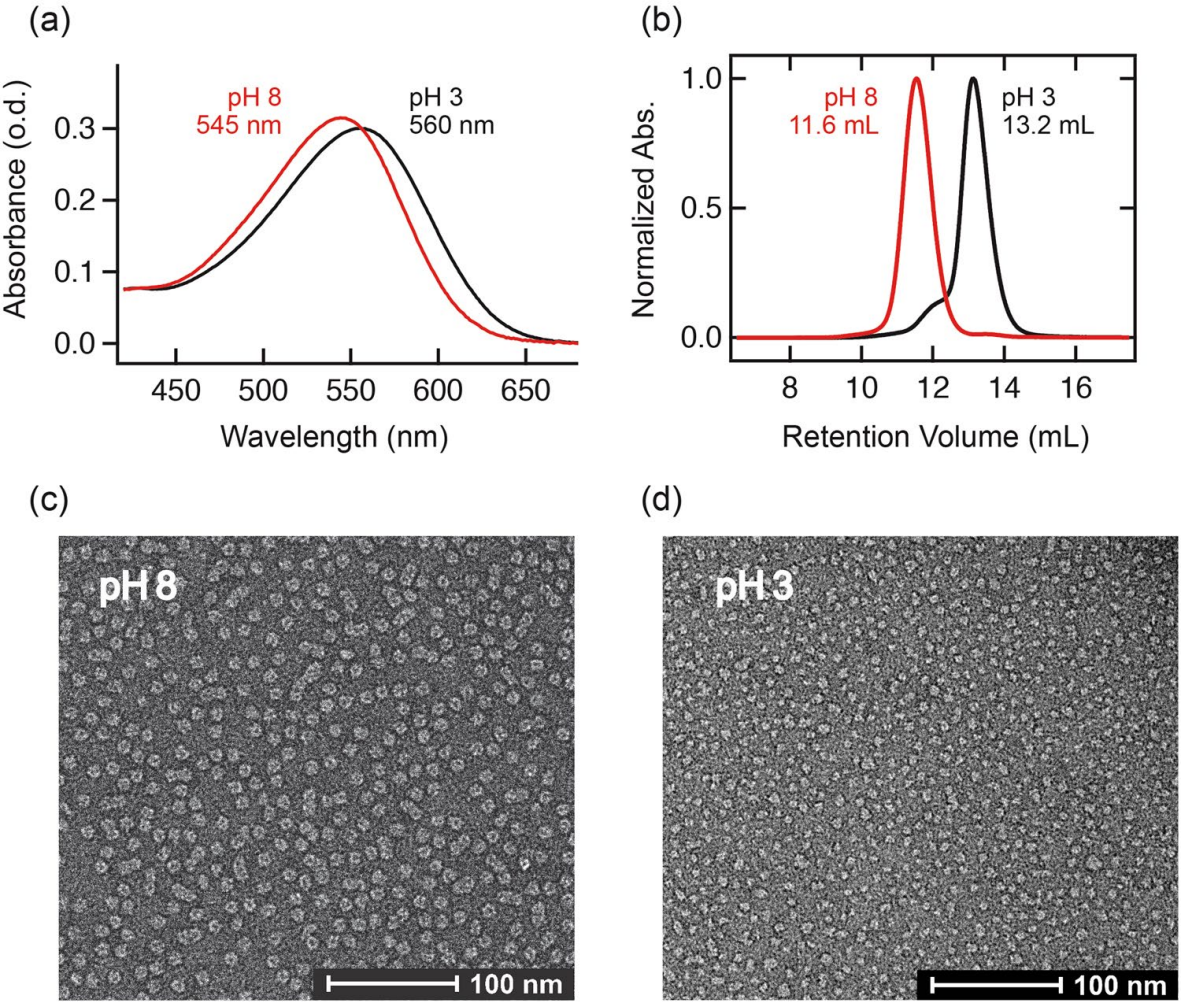
(**a**) UV-Vis spectra of GR at different pH conditions. Absorption maxima are indictaed. (**b**) Size exclusion chromatogram of GR at different pH conditions. Peak elution volumes are indictaed. (**c**,**d**) EM images of GR at pH 8.0 and 3.0, negatively stained with 2% uranyl acetate (Scale bar, 100 nm).

One of the primary methods to characterize microbial rhodopsins is to observe their optical absorption spectrum. In case of BR, solubilization by Triton X-100 produces monomeric BR in solution with a blue-shift of the absorption maximum from 568 to 551 nm^43,44^. In contrast, GPR is known to show a red-shift from 521 to 535 nm when GPR monomers are formed by solubilization with Triton X-100 or OG^36^, but GPR can retain its higher oligomeric state in milder detergent, such as DDM at pH 8.2^19,45^. In addition, GPR shows a pH dependent red-shift from 520 to 540 nm at acidic pH^46^. These results suggest the relationship between oligomeric state and absorption spectral change, probably based on the pKa shift of the retinal counterion. Similar to GPR, GR shows a pH-dependent red-shift of the absorption maximum from alkaline pH (8.0, λ_max_ = 545 nm) to acidic pH (3.0, λ_max_ = 560 nm) in DDM (Fig. 5a). Moreover, we could demonstrate the molecular size change by size-exclusion chromatography (SEC) at different pH (Fig. 5b), consistent with a previous study of GR that showed a pH-dependent oligomer-monomer transition in DDM and GR monomers at acidic pH^25^. At acidic/alkaline pH, the calculated molar masses from the SEC retention volume of the peaks were 87 kDa/239 kDa, respectively. Given that the empty DDM micelle size is ~50 kDa, it is likely that GR (33 kDa) is monomeric at pH 3.0 and becomes oligomeric at pH 8.0. We then performed negative stain electron microscopy (EM) imaging to visualize the oligomer-monomer transition of GR (Fig. 5c,d). The samples collected from SEC peaks at pH 8.0 and 3.0 were directly applied to the carbon-coated EM grids and negatively stained by 2% uranyl-acetate. The images clearly showed homogeneous oligomers at pH 8.0, whereas most of them are dissociated and became roughly one-third in particle size at pH 3.0, consistent with the change of the SEC elution profile due to changes in mass.

### Probing oligomeric states of GR by EPR spectroscopy

Recent comprehensive AFM imaging of microbial rhodopsins showed that GR is pentameric in the reconstituted lipid membrane^17^, although a previous report concluded from SEC that GR has pH-dependent monomer-trimer transition in DDM micelles^25^. To investigate the molecular basis of oligomerization in GR, it is important to clarify if GR can have both trimeric and pentameric states. Therefore, we applied EPR spectroscopy to probe the pH-dependent change of the GR oligomerization state in micelles.

**Figure 6:**
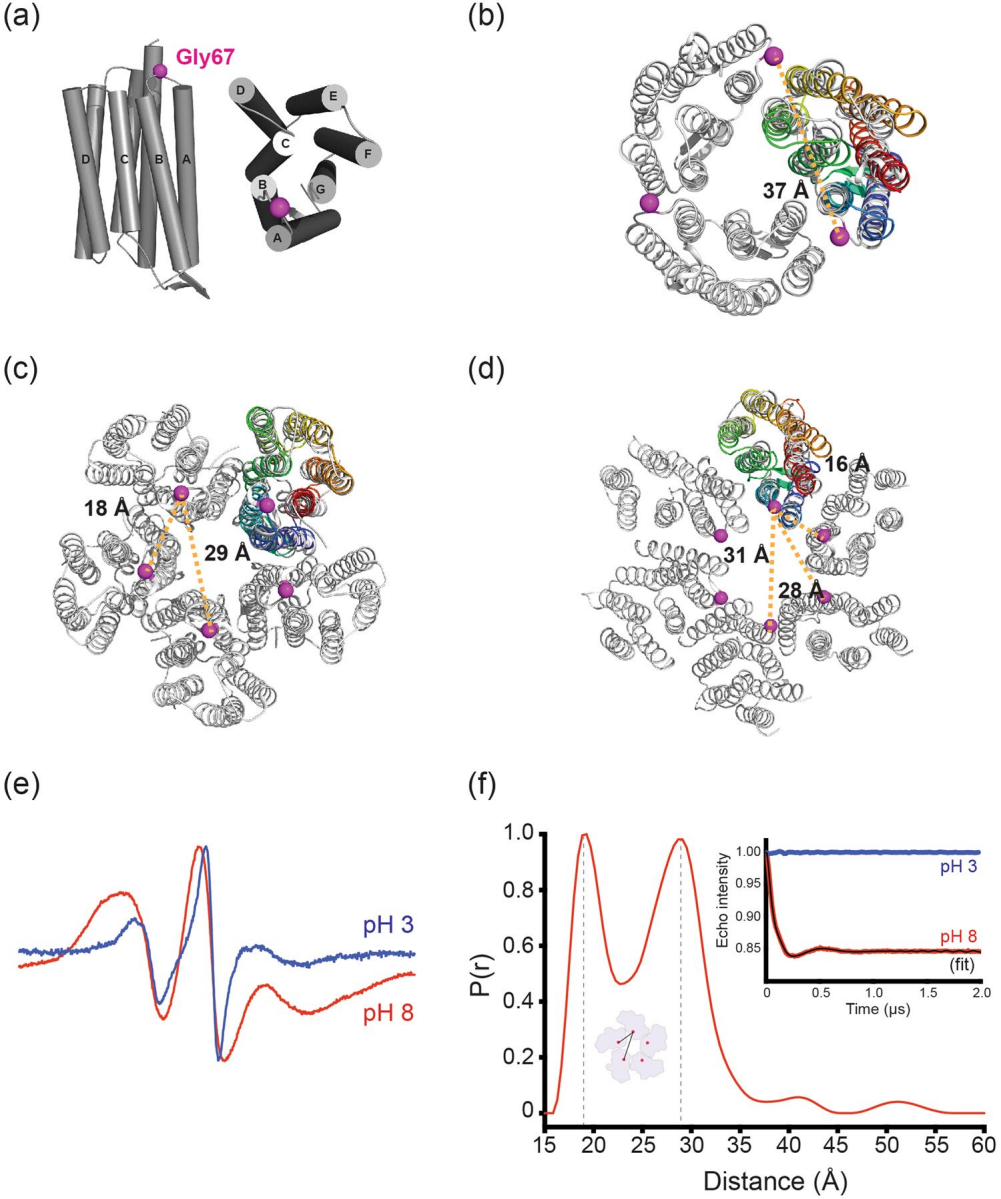
EPR analysis of the oligomeric states of spin labeled GR-67R1. (**a**) Spin-labeling site on GR viewed parallel to the membrane (left) and from the cytoplasmic side (right). The α-carbon of Gly67 is colored magenta and shown as a sphere. (**b**–**d**) Superimposed structure of GR and HsBR (**b**) GR and KR2 (**c**) and BPR (**d**). α-carbon of the residues equivalent to Gly67 in GR are shown as a sphere. (**e**) Room temperature CW EPR spectra of GR-67R1 in 0.05% DDM at pH 3.0 and pH 8.0. Dipolar broadening in the pH 8.0 spectrum is clearly observed. (**f**) DEER distance distribution of GR-67R1 at pH 8.0. Inset: baseline-corrected DEER traces.

To determine the best spin labeling site for intermolecular distance measurement, the crystal structure of GR has been superimposed with the crystal structure of trimeric BR (Fig. 6b), pentameric KR2 (Fig. 6c) and hexameric BPR (Fig. 6d). Gly67 on the cytoplasmic A-B loop has been selected for spin-labeling for EPR/DEER experiments. The spin labeling on site 67 will theoretically yield no distance for a GR monomer, a single ~37 Å distance for a trimer as in BR, two distinguishable distances of ~18 Å and ~29 Å for a pentamer as in KR2, and three ~16 Å, ~28 Å and ~31 Å for a hexamer as in BPR. A single cysteine mutant (G67C) of GR was expressed, purified and collected from SEC peaks at both pH 8.0 and pH 3.0, and then spin labeled to generate a R1 nitroxide side chain. From the continuous wave (CW) EPR line-shape analysis, the samples at both pH 8.0 and pH 3.0 were confirmed to be appropriately spin-labelled. Only at pH 8.0 the CW EPR spectrum is strongly broadened due to the dipolar interaction between nitroxide spin-labels, suggesting a close proximity of the spin labels caused by oligomerization of GR (Fig. 6e). It is suggested by SEC that the majority of GR is monomeric under acidic conditions, and this is also observed in the CW-EPR experiment as the dipolar broadening effects diminished at pH 3.0. The distance between spin-labels was measured by DEER spectroscopy and the oligomeric state of GR was determined accordingly. Figure 6f inset shows the background corrected DEER dipolar evolution functions reflecting the proximity between spin-labels. The sample showed clear DEER signals at pH 8.0, but no signal at pH 3.0, indicating that GR forms oligomer at pH 8.0 and is monomeric at pH 3.0 resulting in no interaction between spins. The DEER signal at pH 8.0 could be further analyzed and two distinct distance peaks at 19 Å and 29 Å were obtained from the distance distribution shown in Fig. 6f. These two distances are in close agreement with the two expected distances from the pentameric model of GR, as shown in the cartoon (Fig. 6c). In an analogous approach, two DEER distance peaks with a similar distance ratio characteristic for pentamers were reported previously for the CorA pentamer^47^. Therefore, we can conclude that the oligomeric state of GR formed at pH 8.0 is pentameric, whereas GR is monomeric at pH 3.0 as shown by the lack of intermolecular distances.

### Oligomerization of microbial rhodopsins

Oligomerization has specific impact on the function of membrane proteins. Microbial rhodopsins are often found as oligomers in native and artificial membranes. Even though many microbial rhodopsins are functional as a monomer, it is unclear why homo-oligomerization would be beneficial for the organism. It has been observed that the trimeric assemblies of BR show greater thermal stability than monomeric BR^48^. Interestingly, the crystal structures of different species, i.e., of blue light-absorbing proteorhodopsin (BPR) show pentameric (HOT75BPR) and hexameric (Med12BPR) arrangements^9^, which is very different from the arrangement of BR trimers. These pentameric/hexameric arrangements of proteorhodopsins are also shown by AFM and NMR studies^17,49^. Specifically, the main cross-protomer interactions occur between residues of helices A, B and C for both pentamers and hexamers in BPR, while the interactions are mainly contributed by helix B and D in BR trimer (Fig. S5). When the interacting helices between protomers of BPR pentamer and hexamer are superimposed, it is obvious that the overall structure of these helices in each protomer is almost identical (Fig. S5d, right half) and only a change of interaction angle by tilting is observed (Fig. S5d, left half). These facts suggest that a pentamer could transform into a hexamer by a small change of interaction, whereas transforming a trimer would require much larger change of protomer organization. Also, the similarity of cross-protomer arrangement between pentamers of sodium pump (KR2)^10,14^ and proton pump (BPR)^9^ suggests that regardless of the function of the pumps, the interacting helices are conserved among the pentameric microbial rhodopsins.

Our DEER-EPR analysis reveals that GR forms pentamers in micelles, which is consistent with a previous AFM and CD analysis in lipid environment^17^. Due to genetic proximity, the GR pentamer model was acquired by superimposing the GR structure onto the KR2 pentamer to find the potential cross-protomer interacting residues between helices A and B (Fig. S6). A histidine residue on helix B (His87) is facing towards the backbone of Ser46 on helix A of the neighboring protomer. As described above, this histidine is well-conserved in both XR-type proton pumps (XR/GR/TR) and proteorhodopsins (BPR/GPR/ESR), paired with aspartate in the same protomer and working as the counterion of the Schiff base. A previous mutation study on GR showed that His87 is the residue titrated by pH change and that breaking the His87-Asp121 interaction at low pH translates into changes of the quaternary structure yielding monomers as the dominant form^25^. Therefore, it is reasonable that His87 plays an important role in the protomer interacting surface. Interestingly, another cross-protomer polar residue pair in this interface near the extracellular surface (Asn43-Asn114 in GR) is conserved in all XR-type rhodopsins (XR/GR/TR and KR2/ClR), but not in proteorhodopsins (BPR/GPR/ESR). The prominent structural differences between these two groups are that the XR-type rhodopsins have a B-C loop with distinct antiparallel β-sheet structure and also share the 3-omega motif, which tethers the B-C loop in the direction of TM helices A and B. The KR2 pentamer found in the crystal and the GR pentamer model strongly suggest the presence of additional interaction between the “flipped” B-C loops of each protomer (Fig. S6) to fix the pentameric organization.

**Figure 7:**
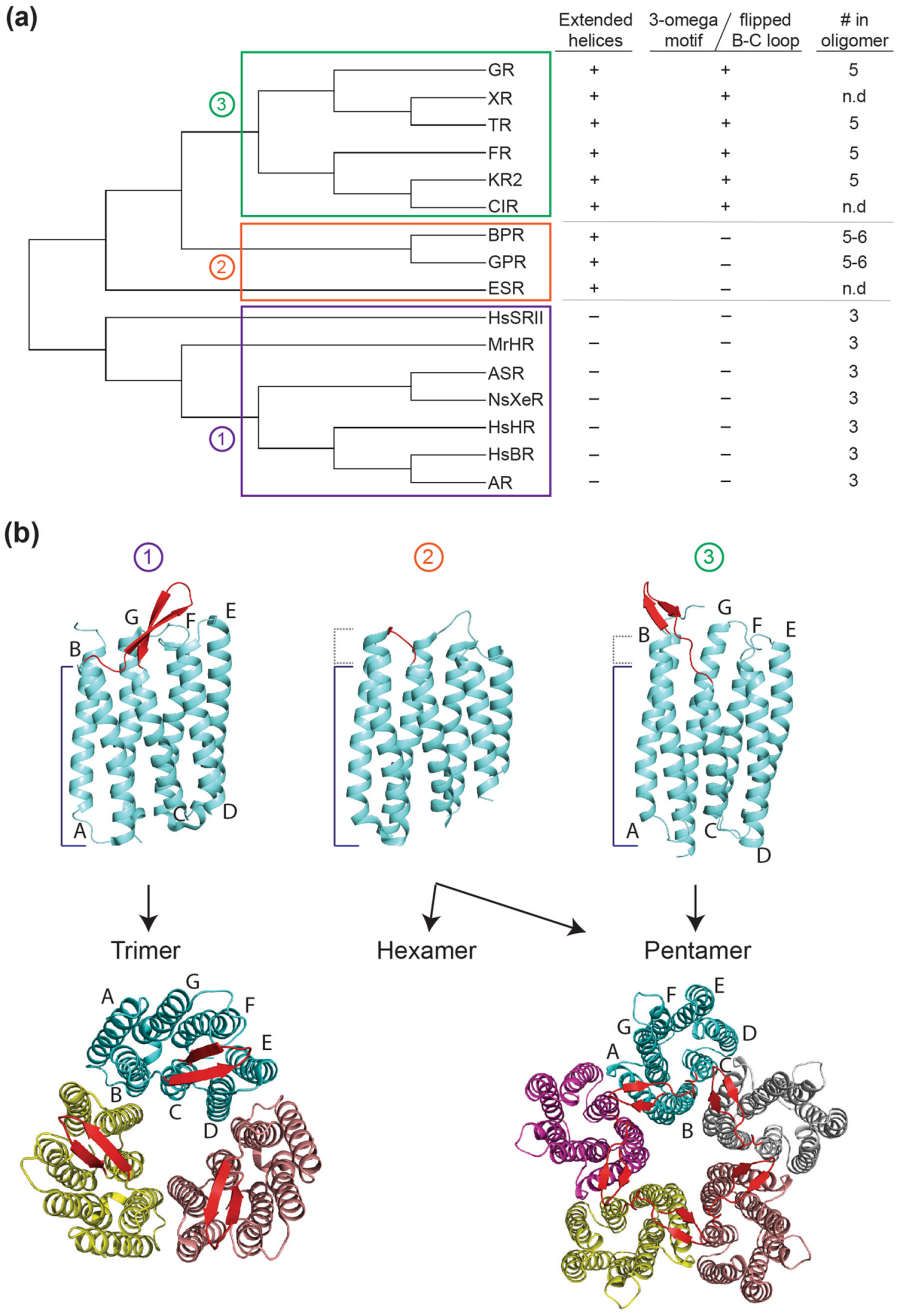
Summary of relationships between the evolutionally altered structural features and oligomerization propensities. (**a**) Phylogenetic tree and structural motifs. The important branches are numbered: extension of the interface helices as 1, acquisition of 3-omega motif and flipped B-C loop as 2 and the functional divergence in XR-type proteins as 3. (**b**) (upper) Crystal structures of BR (left, PDB), BPR-Med12 (middle) and GR (right) viewed parallel to the membrane. (lower) Trimeric organization of BR (left) and modelled pentameric organization of GR (right). B-C loops are colored in Red.

In Fig. 7, a new concept to regroup bacterial pumps based on the relationships between oligomeric states and the conserved structural determinants, i.e. extended helices, 3-omega motif and flipped B-C loop, is summarized. As shown in Fig. 7a, archaeal rhodopsins (AR^50^, BR^18,51^, SR^17^, and HR^17,52^) form trimeric oligomers exclusively, whereas eubacterial rhodopsins form both trimeric (*Anabaena* sensory rhodopsin (ASR)^17,20,53^, xenorhodopsin (XeR)^17,54^ and *M*. *repens* halorhodopsin (MrHR; Besaw, Ou, Morizumi, Vasquez, Miller, Ernst, unpublished observation) and pentameric/hexameric (GR, TR, KR2, FR, BPR, and GPR) oligomers. Therefore, the propensity to form pentamers/hexamers happens to be acquired at the point where *Bacteria* and *Archaea* branched. It is worth noting that ASR, XeR and MrHR have close sequence similarity to archaeal sub-family.

Using available structural data on microbial rhodopsins, we elaborate on the structural determinants causing differences in their oligomerization propensities. First, both archaeal and trimeric eubacterial rhodopsins have relatively short TM helices A, B and G (Fig. 7b, left) compared to extended helices of other non-trimeric eubacterial rhodopsins/proteorhodopsins (Fig. 7b, middle), and XR-type rhodopsins (Fig. 7b, right). Judging from the phylogenetic tree of microbial rhodopsins (Fig. 7a), it is clear that the extension of these helices was acquired at the point where non-trimeric eubacterial rhodopsins (group 2 and 3) branched from trimeric rhodopsins (group 1). Therefore, it is likely that this was a key-event that occurred which converted trimeric to pentameric/hexameric receptors. Second, XR-type rhodopsins have a B-C loop tethered outwards by the 3-omega motif (Fig. 2c) which is formed by an extension of TM helices A and B (group 3: Fig. 7b, right). In contrast, archaeal rhodopsins and trimeric eubacterial rhodopsins have the B-C loop oriented towards the center of the TM helical bundle (group 1: Fig. 7b, left). Interestingly, proteorhodopsins lack the β-sheet structure of the B-C loop as well as the 3-omega motif (group 2: Fig. 7b, middle). Therefore, proteorhodopsins as group 2 (pentameric/hexameric) and XR-type rhodopsins as group 3 (pentameric) receptors were branched by the acquisition of the outwards tethered B-C loop. The KR2 pentamer and our GR pentamer model show that the protomer interface for oligomerization is composed of helices A, B and C, which includes the interaction between B-C loops (Fig. S6). The lack of specific structural motifs at the proteorhodopsin protomer interface might account for their propensity to form hexamers^6,17^ as well as the hexameric crystal structure of BPR. In fact, the hexameric structure of BPR-Med12 (PDB:4JQ6) does not have any interactions within this region, however, the pentameric structure of BPR-HOT75 (PDB:4KLY) has interactions between its long N-terminus and the B-C loop of the neighboring protomer (Fig. S5d).

The reported crystal structure of GR gives insights into structural changes that may occur upon pentamer formation and/or the binding of carotenoid molecules. The high-resolution structure enabled us to confirm the altered hydrogen bonding network in XR-type proton pumps as a common feature, and highlighted candidate residues important for pH mediated oligomerization and proton releasing function. Because both EPR distance measurements and previous AFM results show a pentameric GR state, we find that GR oligomeric states are insensitive to surrounding detergent or lipid environments.

Although further comprehensive characterization would be needed to define the molecular mechanism of oligomerization, there are no known exceptions for the correlation between the direction of B-C loop and the proposed trimer-pentamer oligomerization. This may be essential knowledge for protein engineering of microbial rhodopsins to control oligomeric states which potentially modulate optogenetic functions of the receptors.

## Materials and Methods

### Expression and purification of GR

Wild-type and mutant GR proteins with a 6x histidine tag at the C-terminus were expressed in *E*. *coli* strain C43(DE3) as described previously^39,55^. The cells were induced with 1 mM IPTG for 6 h at 37 °C and all*-trans*-retinal was added to final concentration of 5 µM. The GR proteins were solubilized with 1.0% n-Octyl-*β*-D-glucopyranoside (OG) or n-dodecyl-*β*-D-maltoside (DDM) at room temperature followed by ultracentrifugation at 100,000 × g to remove unsolubilized material. For GR purification, the supernatant was loaded onto a TALON Co^2+^ column in the solubilizing buffer and eluted by lowering pH. GR fractions were further purified on a Superdex 200 10/300 GL column with running buffer (20 mM HEPES, pH 7.5, 300 mM NaCl containing 1.0% OG or 0.04% DDM). The peak fraction of GR was collected and used immediately without freezing. The UV-vis absorption spectrum was measured using a CARY 60 UV-Vis spectrophotometer equipped with a Peltier temperature control unit (Agilent Technologies) at 15 °C.

### Crystallization of GR

GR crystals were grown in bicelles using the hanging drop method similar to what was previously described for bacteriorhodopsin crystallization^56,57^. The purified GR in 1% OG was concentrated to ~15 mg/mL and mixed with 24% (w/v) DMPC:CHAPSO bicelles in a 1:2 ratio (final 8% bicelles) on ice where the mixture remains liquid and kept for 30 min to equilibrate. Crystallization was initiated by mixing 4 µL of the protein/bicelle mixture with 1.4 µL of well solution (2.6–2.8 M NaH_2_PO_4_ pH 3.4, 180 mM 1,6-hexanediol, 3.5% triethylene glycol and 40 mM zinc acetate) and 0.6 µL of 1.0% OG in water. The crystallization trays were then placed in an incubator at 34 °C to allow bicelles to form gel-like phase, and square shaped thin crystals appeared within a few days. To grow thicker crystals, the coverslips were transferred onto wells with solution containing slightly lowered precipitant concentration (2.4–2.6 M NaH_2_PO_4_) to enhance the lateral growth of crystals for one more week. Crystals were then harvested and flash frozen in liquid nitrogen after being soaked in the well solution containing 10% ethylene glycol as a cryoprotectant. For Raman spectroscopy, crystals were independently harvested without freezing and diluted with well solution containing 3.0 M NaH_2_PO_4_.

### Data collection and structure determination

X-ray diffraction data were collected at beamline 23ID-D of the Advanced Photon Source (APS) synchrotron at 100 K, with a Pilatus 6 M detector. The crystals were exposed to a 20-μm-wide, 20-μm-high beam for 0.2 s at 0.2° oscillation/frame with no attenuation at a wavelength of 1.0331 Å. Diffraction images from three crystals were processed using the software package available at the beamline and merged by Aimless in the CCP4 software package. The structure of XR (PDB entry 3DDL) was used as a search model for molecular replacement with Balbes^58^, and the model was subjected to maximum-likelihood refinement with Refmac5 with manual model building using Coot in the CCP4 software package and further refinement using Phenix^59,60^. The data-collection and refinement statistics are summarized in Table 1.

### Size exclusion chromatography

Size exclusion chromatography was used for both preparative and analytical purposes. All runs were performed using an ÄKTA purifier with a Superdex 200 10/300 GL gel filtration column (GE Healthcare). Briefly, for the oligomerization analysis, the column was equilibrated with a buffer containing either 20 mM Tris-HCl pH 8.0, 300 mM NaCl and 0.04% DDM, or 20 mM sodium citrate pH 3.0, 300 mM NaCl and 0.04% DDM.

### Negative-stain EM imaging

A droplet of 3.5 µL of purified GR at a concentration of around 0.05 mg mL^−1^ was applied to glow-discharged carbon coated copper grids (TED PELLA, CAT# 01840-F) and negatively stained with 2% uranyl acetate. Images were collected on a Talos L120C transmission electron microscope at the Microscopy Imaging Laboratory at the University of Toronto.

### Raman spectroscopy

Raman spectroscopy of GR crystals was performed using FRA106/s accessory of the Bruker IFS66vs spectrometer, with Nd-YAG laser excitation at 1064 nm, at a 2 cm^−1^ resolution. Five microliters of crystal suspension in the crystallization buffer was placed in a metallic holder, and 7000 spectra were averaged. Spectrum of the crystallization buffer was recorded under the same conditions and subtracted from the crystals’ spectrum.

### Time-resolved laser spectroscopy

Time-resolved laser spectroscopy in the visible range (flash-photolysis) was performed using a custom-built single-wavelength spectrometer described previously^61^. The GR crystal suspension was diluted with crystallization buffer to produce an optically transparent sample and the photocycle was initiated with 7 ns pulses of the second harmonic of an Nd-YAG laser at 532 nm (Continuum Minilite II, Energy density at the sample ~2 mJ/cm^2^). Absorption changes of the monochromatic light (provided by an Oriel QTH source and two monochromators) were followed using an Oriel photomultiplier, an amplifier with a 350 MHz bandwidth, and a Gage AD converter (CompuScope 12100-64 M). The kinetic traces were averaged (1000 traces) and converted into a quasi-logarithmic time scale using in-house software^62^.

### Site-directed mutagenesis and spin-labeling of GR mutant

Wild-type GR has no reactive cysteine residues for spin-labeling. Hence, a G67C mutation was introduced by PCR using a QuikChange Lightning (Agilent) mutagenesis kit. The site G67 was selected based on the fact that cysteine replacement and spin labeling of the corresponding residue in BPR (S55) had no influence on the multimerization of BPR^45^. The sequence of the mutant was confirmed by DNA sequencing (ACGT Corporation, Toronto, ON). Purified GR mutant G67C was mixed with 100 μM of 1-oxyl-2,2,5,5-tetramethyl-∆3-pyrroline-3-methyl methanethiosulfonate (Toronto Research Chemicals) and incubated at room temperature for 30 min to form spin-labeled GR-67R1. The protein stability and pH-dependent oligomerization of the G67C mutant were confirmed by absorbance spectra and SEC peak profiles throughout the sample preparation process. Excess spin label was removed, and buffer was exchanged to that with desired composition using a 30 kDa MWCO concentrator (Amicon).

### CW-EPR measurements

X-band continuous wave (CW)-EPR data of the spin-labeled GR-67R1 at different buffer compositions were acquired using a Bruker ELEXSYS E500 spectrometer equipped with a Bruker EP 041 MR microwave bridge. Samples were loaded into 0.6 mm ID and 0.84 mm OD capillaries and inserted into an ER 4123D dielectric resonator for measurement. The field sweep for data collection was 100-G, modulation amplitude was 2-G, and the incident microwave power was 0.5029 mW. Data sets were averages of 30 scans. Each CW-EPR measurement was acquired at room temperature.

### DEER measurements

For DEER measurements, deuterated glycerol was added to the samples as a cryo-protectant (final concentration 20%). Spin-labeled GR-67R1 was loaded into quartz capillaries (1.5 mm ID and 1.8 mm OD) and flash frozen using a dry ice/ethanol bath. After freezing, the capillaries were loaded into an ER 5107D2 Q-band flexline resonator and Q-band measurements were performed at 80 K on a Bruker Elexsys 580 spectrometer with a Super Q-FTu Bridge. A 32-ns π-pump pulse was applied to the low field peak of the nitroxide field swept spectrum, and the observer π/2 (16 ns) and π (32 ns) pulses were positioned 50 MHz (17.8 G) upfield, which corresponds to the nitroxide center line. Distance distributions were obtained from the raw data using the LabVIEW (National Instruments) program “LongDistances” [developed by Christian Altenbach and Wayne Hubbell, University of California, Los Angeles (UCLA)] that can be downloaded from http://www.biochemistry.ucla.edu/biochem/Faculty/Hubbell/. The DEER distance peak simulations were conducted with the open-source package Multiscale Modeling of Macromolecules (MMM)^63^.

### Accession number

Coordinates and structure factors for *Gloeobacter* Rhodopsin have been deposited in the Protein Data Bank with accession number 6NWD.

## Supplementary information

Supplementary Information

## Data Availability

The datasets generated during and/or analysed during the current study are available from the corresponding author on reasonable request.
