# Supplementary material for: X-ray Crystallographic Structure and Oligomerization of Gloeobacter Rhodopsin
Source: Sci Rep. 2019 Aug 2;9:11283. doi: 10.1038/s41598-019-47445-5 (PMC6677831; doi:10.1038/s41598-019-47445-5)
Supplement: Supplementary file 1 — Supplementary Information [file 41598_2019_47445_MOESM1_ESM.pdf]

Supplementary Information

X-ray Crystallographic Structure and Oligomerization of *Gloeobacter* Rhodopsin

Takefumi Morizumi[1*], Wei-Lin Ou[1*], Ned Van Eps[1], Keiichi Inoue[3], Hideki Kandori[4,5], Leonid S. Brown[6], Oliver P. Ernst[1,2]

[1] Department of Biochemistry, University of Toronto, Toronto, Ontario M5S 1A8, Canada

[2] Department of Molecular Genetics, University of Toronto, Ontario M5S 1A8, Canada

[3] The Institute for Solid State Physics, University of Tokyo, Kashiwa, Chiba 277-8581, Japan

[4] Department of Life Science and Applied Chemistry, Nagoya Institute of Technology, Showa-ku, Nagoya 464-8555, Japan

[5] OptoBioTechnology Research Center, Nagoya Institute of Technology, Showa-ku, Nagoya 464-8555, Japan

[6] Department of Physics, University of Guelph, Guelph, Ontario N1G 2W1, Canada

*Both authors contributed equally to this work

Corresponding author: Oliver P. Ernst (oliver.ernst@utoronto.ca)

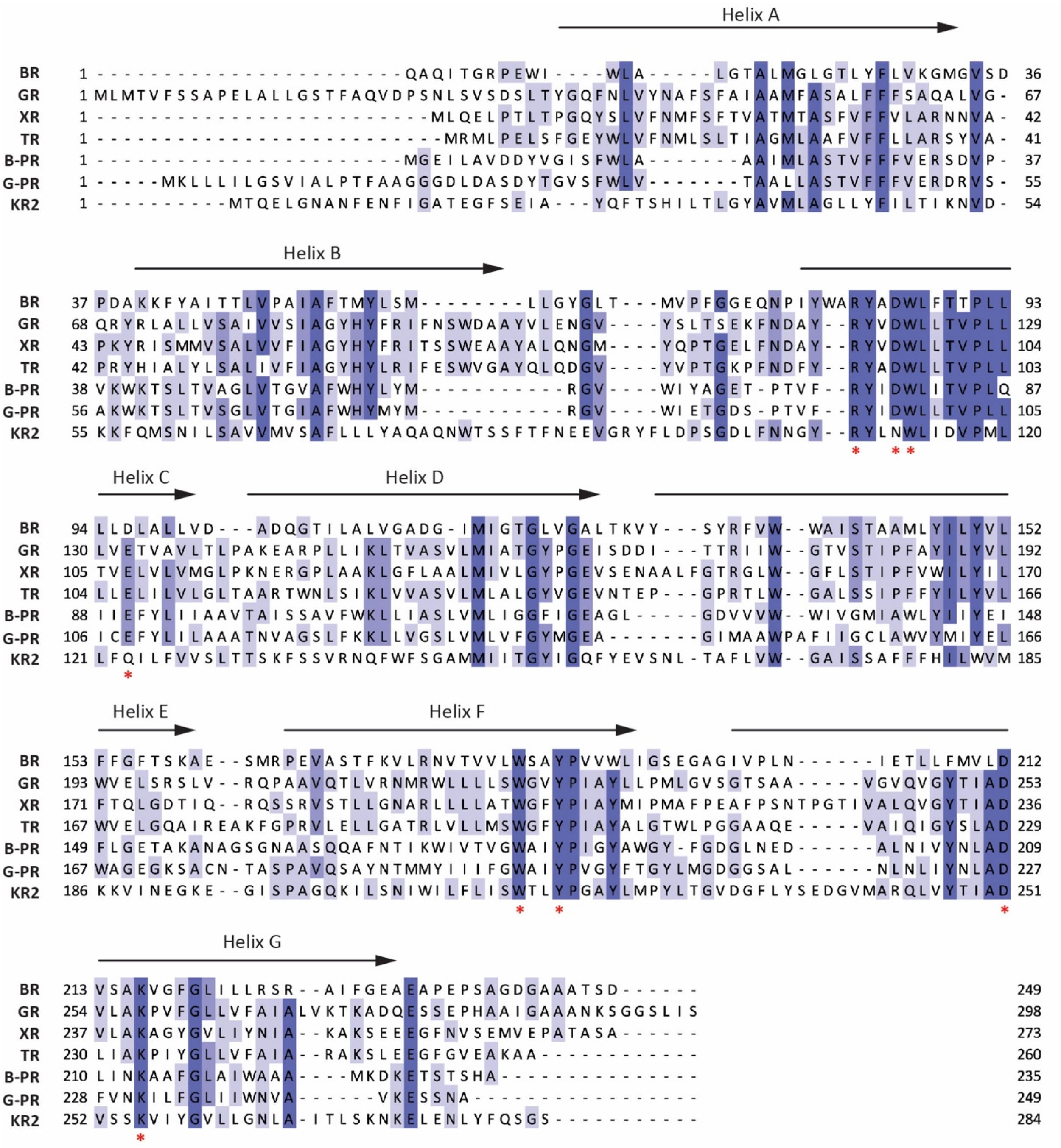

Supplementary Figure S1. Amino acid sequence alignment of microbial rhodopsins. Alignment of sequences of BR (WP_010903069.1), GR (WP_011140202.1), XR (WP_011404249.1), TR (WP_014629850.1), B-PR (PDB entry 4JQ6), G-PR (UniProt Q9F7P4.1), and KR2 (PDB entry 3X3B).

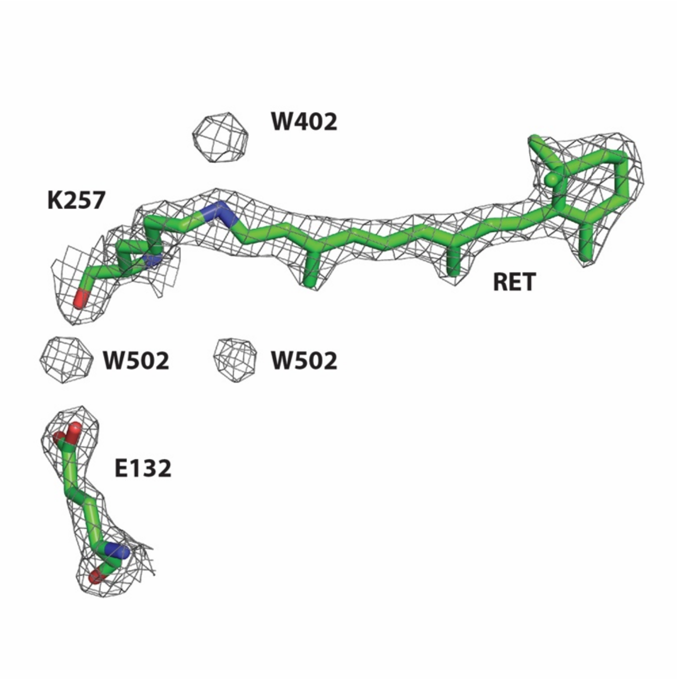

Supplementary Figure S2. 2*Fo-Fc* electron density map of retinal Schiff base region including water molecules contoured at 2.0 σ.

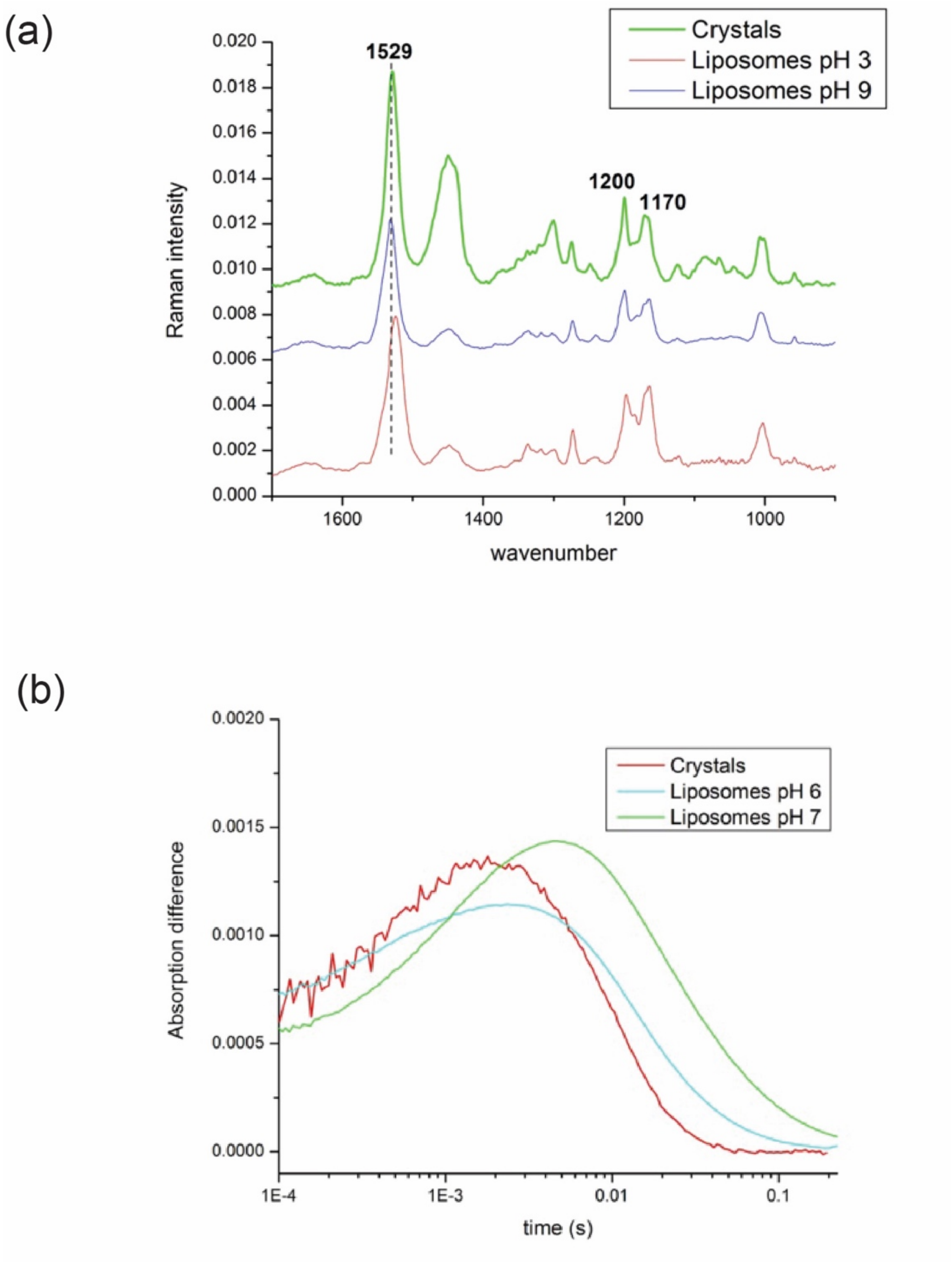

Supplementary Figure S3. (**a**) Retinal conformation and environment of crystalline GR probed by Raman spectroscopy. Raman spectrum of crystalline GR (green) compared with the spectra of lipid-reconstituted GR at pH 9 (blue) and pH 3 (red) obtained previously (Ref. 39). (**b**) Photocycle kinetics of crystalline GR. Kinetics of laser-induced absorption change of GR crystals (red) measured at 620 nm in comparison with those obtained previously for lipid-reconstituted GR at pH 6 (cyan) and pH 7 (green).

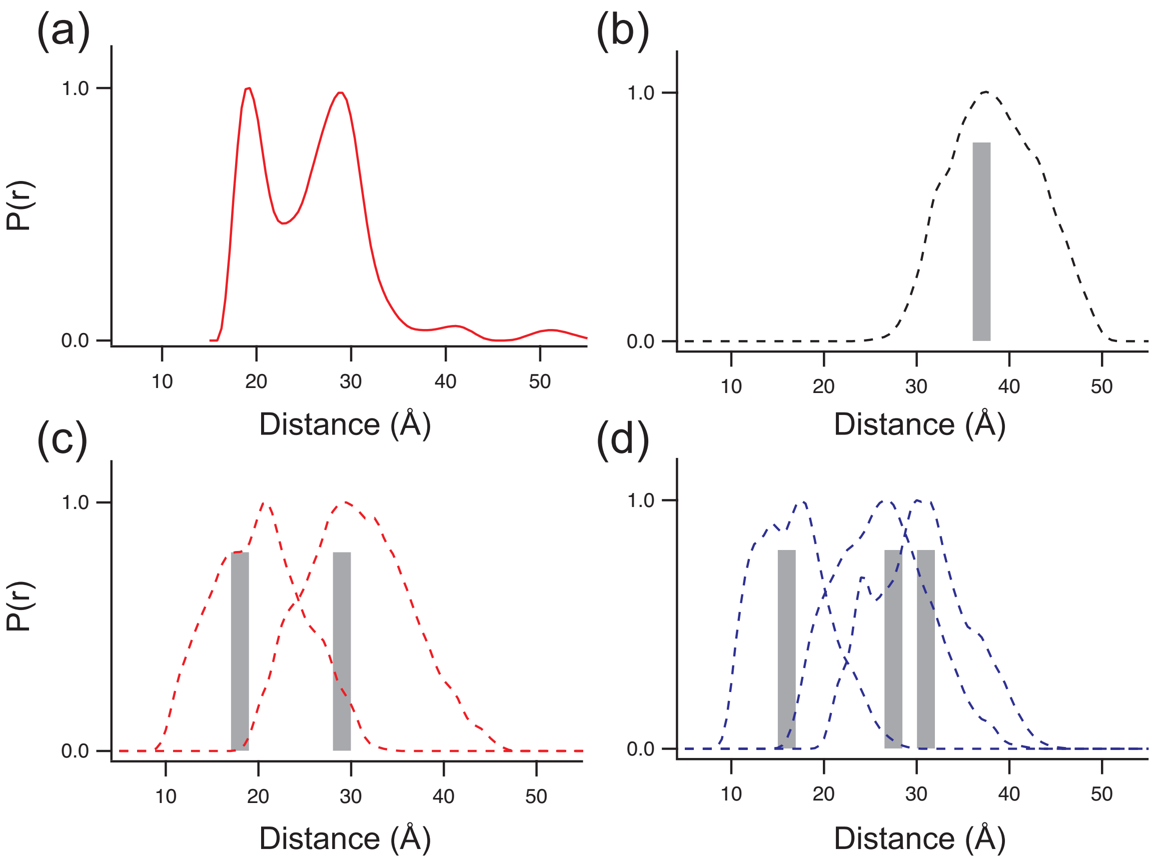

Supplementary Figure S4. DEER distance simulations of GR oligomers conducted with Multiscale Modeling of Macromolecules software (MMM; Ref. 63). (**a**) DEER distance distribution obtained from GR-67R1 experimental data. (**b-d**) MMM modeling. Broken curves are the simulated distance probabilities considering rotamers of spin-labels for (**b**) trimer, (**c**) pentamer and (**d**) hexamer arrangements of GR, respectively. Trimer, pentamer and hexamer arrangements were taken from PDB entries 4XXJ, 4XTO, and 4JQ6, respectively. For MMM analysis the R1 spin label was modeled at sites corresponding to G67 of GR. Grey bars indicate the distances between the alpha carbons at sites corresponding to G67 of GR.

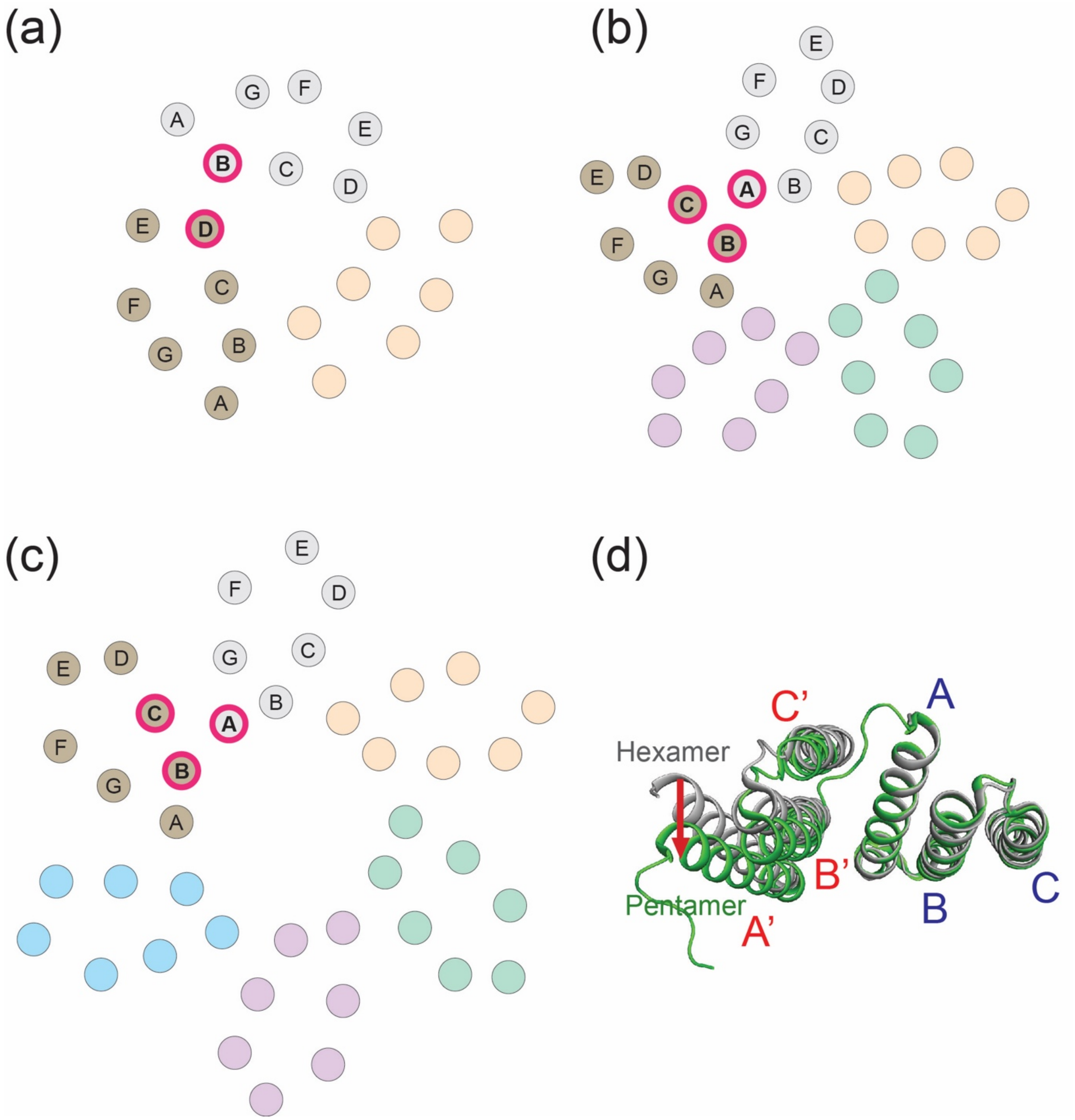

Supplementary Figure S5. Oligomeric organization of microbial rhodopsin TM helices**.** Cross-sectional views of helical arrangements of BR trimer (**a**), KR2 pentamer (**b**), and BPR-Med12 hexamer (**c**) shown from the extracellular side. (**d**) The cross-protomer helices A, B and C in BPR-Med12 hexamer (PDB entry 4JQ6, gray) and BPR-HOT75 pentamer (PDB entry 4KLY, green) were superimposed, yielding the relative orientation change of helices A', B' and C' in the neighbouring protomers due to the different oligomeric arrangements.

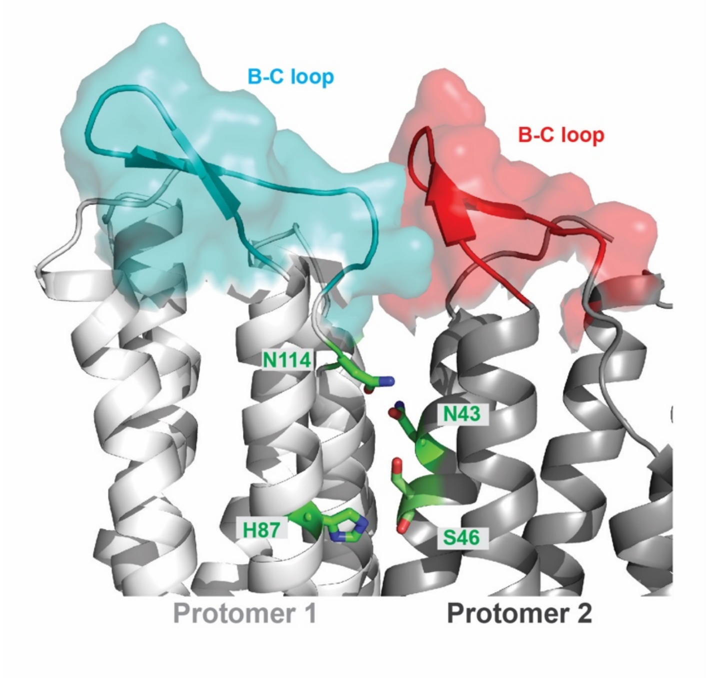

Supplementary Figure S6. Potential cross-protomer interface of GR derived from superimposition of GR structure and the crystal structure of KR2. Side chains of Asn114 and His87 on helix B of protomer 1 and Ser46 and Asn43 on helix A of protomer 2 are shown as sticks. The protein surfaces of B-C loops are shown as colored surfaces to show the potential interaction between loops.
